# Nerve pathologic features differentiate POEMS syndrome from CIDP

**DOI:** 10.1186/s40478-016-0389-1

**Published:** 2016-10-31

**Authors:** Ezequiel A. Piccione, Janean Engelstad, Peter J. Dyck, Michelle L. Mauermann, Angela Dispenzieri, P. James B. Dyck

**Affiliations:** 1 University of Nebraska Medical Center, 988435 Nebraska Medical Center, Omaha, NE 68198-8435 USA; 2 Mayo Clinic, Department of Neurology, 200 First Street SW, Rochester, MN 55905 USA; 3 Mayo Clinic, Department of Medicine, Division of Hematology, 200 First Street SW, Rochester, MN 55905 USA

**Keywords:** POEMS, CIDP, Neovascularization, Demyelination, VEGF

## Abstract

The objective of this study is to determine if the nerve pathology in patients with POEMS syndrome is different from CIDP. We hypothesized that nerve biopsies from patients with POEMS syndrome would have more small vessels and axonal degeneration but less inflammation than CIDP.

We performed a retrospective analysis of nerve biopsies performed on “classic” CIDP and POEMS cases. Nerve biopsies were blinded and reviewed by two of the authors (EAP, PJBD). Teased fibers, paraffin-embedded sections, semithin sections and immunostains were analyzed. Small endoneurial and epineurial vessels were counted on paraffin sections with smooth muscle actin (SMACTIN) preparation to judge for neovascularization.

A total of 61 cases (35-POEMS, 26-CIDP) were included. The POEMS-group had significantly higher axonal degeneration and fewer normal myelinated fibers on teased fiber preparations. The CIDP-group had significantly more endoneurial mononuclear inflammation on paraffin sections and immunostains. Large onion-bulbs were present only in CIDP cases. A significantly higher number of epineurial vessels was present in POEMS biopsies, with a total count of 120 epineurial vessels predicted as best cutoff to differentiate both conditions (77 % specific and 54 % sensitive).

In conclusion, nerve biopsy can be helpful in distinguishing POEMS syndrome from CIDP. POEMS syndrome demonstrates more axonal degeneration and epineurial neovascularization whereas CIDP has greater endoneurial inflammation and onion-bulb formation. These findings support the idea that there are differing underlying mechanisms for these disorders, POEMS being related to paraneoplastic vasculopathy associated with angiogenic factors and CIDP related to inflammatory demyelination.

## Introduction

The peripheral neuropathy of POEMS Syndrome (polyneuropathy, organomegaly, endocrinopathy, M-protein, and skin changes) has great similarities with that of CIDP (chronic inflammatory demyelinating polyradiculoneuropathy). Both neuropathies cause a motor and sensory, chronic and progressive neuropathy, with polyradicular features (distal and proximal involvement), and with demyelinating electrophysiological features. Because of these similarities, distinguishing POEMS syndrome from CIDP can be challenging and many cases of POEMS cases are initially misdiagnosed as CIDP [1]. Most of the patients in our own clinical practice whom we eventually diagnosed with POEMS syndrome were initially misdiagnosed as having CIDP. Because both conditions are treatable and because the treatments for them are different, it is important to get the diagnosis correct as early as possible to ensure good treatment outcome. Consequently, it may be clinically useful to consider differences in the clinical pattern, electrophysiological findings and pathological findings, which can help distinguish the two disorders. While the skin and bone changes are informative of the POEMS syndrome, early differentiation may be difficult. Here, we asked if pathological alterations could be a useful approach to recognize differences between these two disorders. We conducted a large retrospective review of nerve biopsies performed at our institution from patients with POEMS syndrome and from patients with CIDP and regraded them in a blinded fashion to determine if numbers of small epineurial vessels and findings of inflammatory demyelination were useful in separating these disorders.

## Materials and methods

After Institutional review board approval, we queried “CIDP” and “POEMS syndrome” in the Mayo Clinic-Rochester database from 1979 to 2013. Over 300 patients records were reviewed. “Classic” CIDP cases were included only. Clinical criteria required progressive or fluctuating course over ≥ 8 weeks, preferential impairment of large fibers and proximal and distal involvement of 4 limbs with hypo or areflexia [2]. Only if the clinical criteria were met, nerve conductions and EMG were reviewed. Mayo Criteria for demyelination was used [3]. Electrophysiologic criteria was met if:

1). Conduction block or temporal dispersion was present in one motor nerve and in at least one other motor nerve there were 2 or more of the following: prolonged F-waves, prolonged distal latency, slowing of conduction velocity. 2). No conduction block or temporal dispersion was found but in at least 2 motor nerves there were two or more of the demyelinating features described above. Supportive features of diagnosis were the 1) CSF analysis (protein level >60 mg/dL with leukocyte count <10 cells/_L) and 2) response to immunosuppressive therapy. Cases of CIDP included in this study were “definite” (fulfilled all clinical and electro-physiologic criteria) or “probable” (fulfilled all clinical criteria and at least one of the supportive features). These inclusion criteria were used in a prior CIDP publication [4].

POEMS cases were included if they had polyneuropathy and monoclonal plasma cell disorder, one other major and one minor Dispenzieri criteria [5].

All patients that met criteria for CIDP or POEMS syndrome and had a nerve biopsy taken were included in our study. Only standard distal whole cutaneous sensory nerve biopsies (mostly sural) were included (not proximal fascicular) so as to use the normative data from our laboratory.

Nerve biopsies from these patients were retrieved and blinded to the diagnosis by one of the authors (J.E) and reviewed by two other authors (E.A.P. and P.J.B.D.). The biopsies were labeled with age and gender, which was the only information available to both reviewers. Teased fibers were analyzed and re-graded according to previously published criteria [6]. Transverse and longitudinal paraffin sections stained with Masson’s trichrome, hematoxylin and eosin (H&E) and luxol-fast-blue (LFB) were analyzed. Immunohistochemistry preparations included leukocyte common antigen [LCA, CD45] and macrophage (KP-1, CD68). Semithin epoxy sections stained with methylene blue and p-phenylenediamine were reviewed.

The pathologic alterations were graded semi-quantitatively: inflammatory cell collections were documented as small collections (10–49 cells), moderate collections (50–99 cells), and large collections (>100 cells). The location of inflammation (endoneurial, perineurial and epineurial) was also recorded. Onion-bulbs (OB) were considered present if they were at least moderate (3–4 layers) or large (>5 layers) in size as rudimentary or small onion bulbs can appear in most chronic polyneuropathies. Nerves were graded for myelinated fiber loss (normal, mild, moderate and severe) and distribution of fiber loss (generalized or multifocal).

Additional slides were immunostained with anti-human smooth muscle actin (SMACTIN). Manual count of small endoneurial and epineurial vessels (neovascularization) was done on one transverse section on one slide per each biopsy. Blood vessels from each section were counted twice for better precision. After all pathologic findings had been documented, biopsies were unblinded (by J.E) and the data was analyzed.

Due to sample size differences and non-normal distribution of the data, nonparametric statistical comparisons were performed. The Mann–Whitney Test (Wilcoxon) was used to examine comparisons of continuous variables between the two groups. Pearson’s chi-squared test was used for comparisons of categorical variables. All statistical tests were two-tailed and *P* < 0.05 was considered significant for all comparisons. JMP software for MAC (SAS institute) and SPSS software (SPSS Inc, Chicago, IL) were used for all statistical analyses.

## Results

A total of 61 cases were included in the study with a median age of 53 years and a range of 14–85. The POEMS group included 35 biopsies (median age 49, range of 35–76) and the CIDP group 26 biopsies (median age 64, range of 14–85). There was a male predominance in the POEMS group (22 males and 13 females, M/F ratio of 1.7:1) and a slight female predominance in the CIDP group (12 males and 14 females, M/F ratio of 1:1.2).

**Fig. 1:**
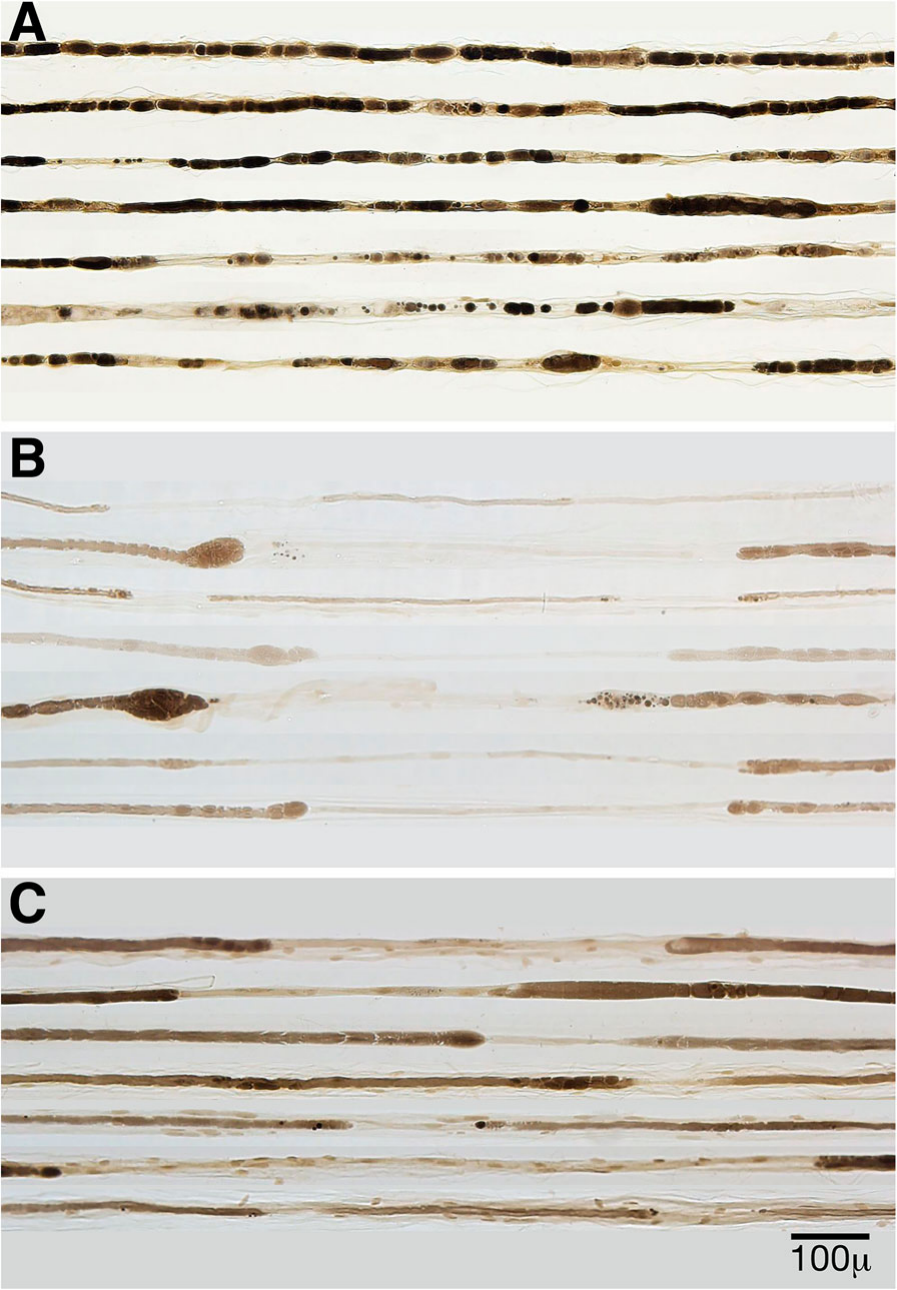
Teased nerve fiber preparations in POEMS (**a** and **b**) and CIDP biopsies (**c**). Increased rates of segmental demyelination and remyelination are present to similar degrees in both POEMS (**b**) and CIDP (**c**) biopsies. Whereas there was significantly greater degrees of axonal degeneration present in POEMS (**a**) biopsies

Analysis of teased fibers preparations (available for 60/61 cases, Table 1) revealed a higher percentage of normal fibers in the CIDP group overall. POEMS cases demonstrated significantly higher (*P* < 0.01) rates of axonal degeneration (Fig. 1a): severe rates of axonal degeneration (20–50 % of teased fibers) were more in common in POEMS syndrome (16 cases vs. 4 in CIDP group). Demyelination was abnormal in both groups (Fig. 1b-c). A moderate degree of demyelination on teased fibers (5–20 %) was common in both conditions (15 POEMS and 12 cases of CIDP) and the percentages were not significantly different between the two groups.

**Table 1:** Teased nerve fiber results for POEMS and CIDP biopsies

Teased fiber condition	POEMS		CIDP		*P* value
	Median	Range	Madian	Range	
Normal A, B (%)	56	9–76	74	16–93	<0.01*
Demyelination: C, D (%)	9	1–31	8	1–62	=0.7
Remyelination: F, G (%)	9	3–33	9	2–23	=0.5
Axonal: E, H (%)	22	3–73	7	1–55	<0.01*
Classifiable (no.)	81	11–105	70	22–100	=0.5
Empty (no.)	17	4–75	20	2–85	=0.6

Results from classifiable fibers are expressed in percentages and unclassifiable fibers (empty strands) are expressed in numbers. *P* value <0.05 were considered significant and are marked with a star (*) and bolded text

**Fig. 2:**
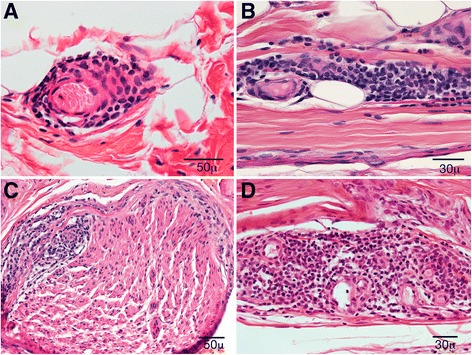
Cross section (**a** and **c**) and longitudinal (**b** and **d**) paraffin section stained with H&E showing inflammatory infiltrates in POEMS and CIDP biopsies. In POEMS biopsies, the inflammation was mostly epineurial perivascular (**a** and **b**) whereas in CIDP the inflammation was endoneurial perivascular (**c**) and epineurial perivascular (**d**)

Paraffin sections analysis revealed that the CIDP group had significantly higher (*p* < 0.01) degree of endoneurial mononuclear inflammation (Fig. 2). Small collections of endoneurial inflammation were commonly encountered in CIDP. There were 2 CIDP cases (but none of POEMS) that showed moderate endoneurial collections (Fig. 2c). Presence of epineurial inflammation was not different overall between the two groups – there were small collections frequently seen in both groups (14 CIDP and 12 POEMS cases). However, the one case with large epineurial collections occurred in a CIDP biopsy (Fig. 2d).

Immunostains for CD-45 and CD-68 were available in 41/61 cases. There was a significantly higher (*P* = 0.04) degree of endoneurial CD-45 positivity in CIDP biopsies. This finding was consistent with the increased endoneurial inflammation seen on paraffin sections. There was no clear difference in endoneurial or epineurial CD-68 (macrophage) staining between the two groups.

**Fig. 3:**
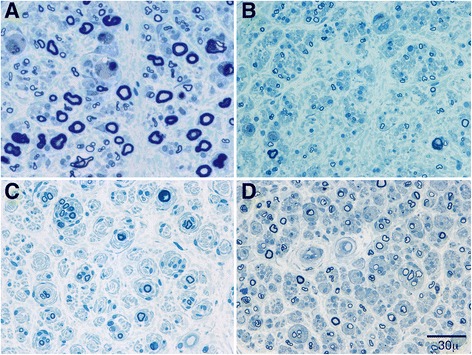
Semithin epoxy transverse section stained with methylene blue from POEMS (**a** and **b**) and CIDP (**c** and **d**) biopsies. The biopsies show decreased density of myelinated fibers, active axonal degeneration and lack of onion-bulbs in the POEMS (**a** and **b**) and multiple large onion-bulbs, decreased number of large myelinated fibers and regenerating clusters present in the CIDP biopsies (**c** and **d**)

CIDP showed more frequent and larger OB formation (*P* < 0.01, Fig. 3). Four cases of CIDP and no cases of POEMS demonstrated frequent OB formation (*P* < 0.01). In addition, moderate and large OB were only present in six cases of CIDP and in no cases of POEMS (*P* < 0.01).

**Fig. 4:**
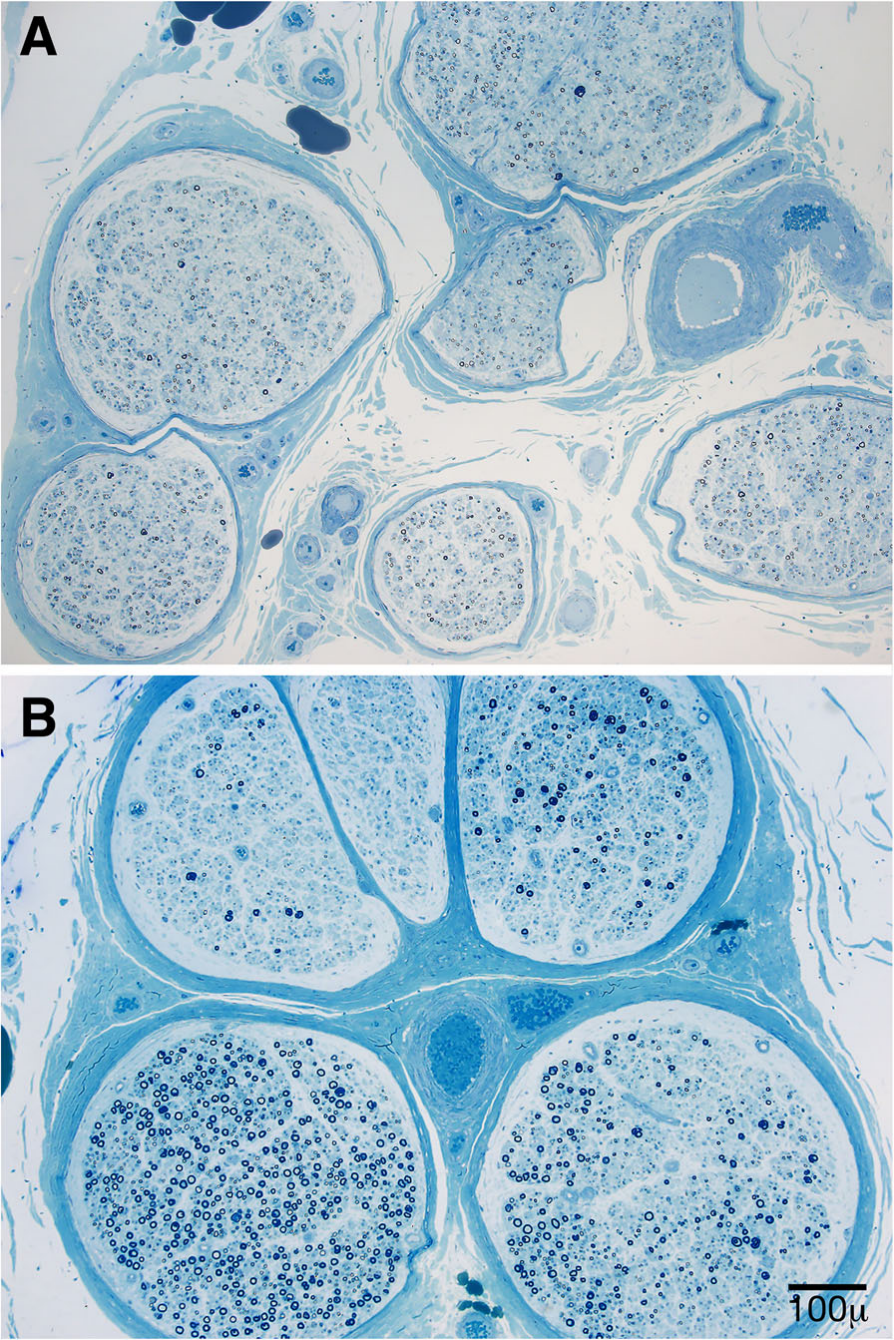
Semithin transverse epoxy sections stained with methylene blue from POEMS (**a**) and CIDP (**b**) biopsies. Diffuse loss of myelinated fibers was typical of biopsies in POEMS syndrome (**a**) whereas multifocal loss of myelinated fibers was commonly found in CIDP (**b**) biopsies

In semithin epoxy sections, the myelinated fiber density was significantly more reduced in the POEMS cohort (*p* = 0.01) than in the CIDP cohort. Moderate and severely decreased fiber densities were present in 17 cases of POEMS and 8 cases of CIDP. The patterns of fiber loss were significantly different between the two groups (Fig. 4). Diffuse fiber loss was more common (*P* < 0.01) in the POEMS group (31/35) than in CIDP (12/26). Multifocal fiber loss was more common (*P* = 0.048) in CIDP (6/26 cases) than in POEMS (2/31 cases).

**Fig. 5:**
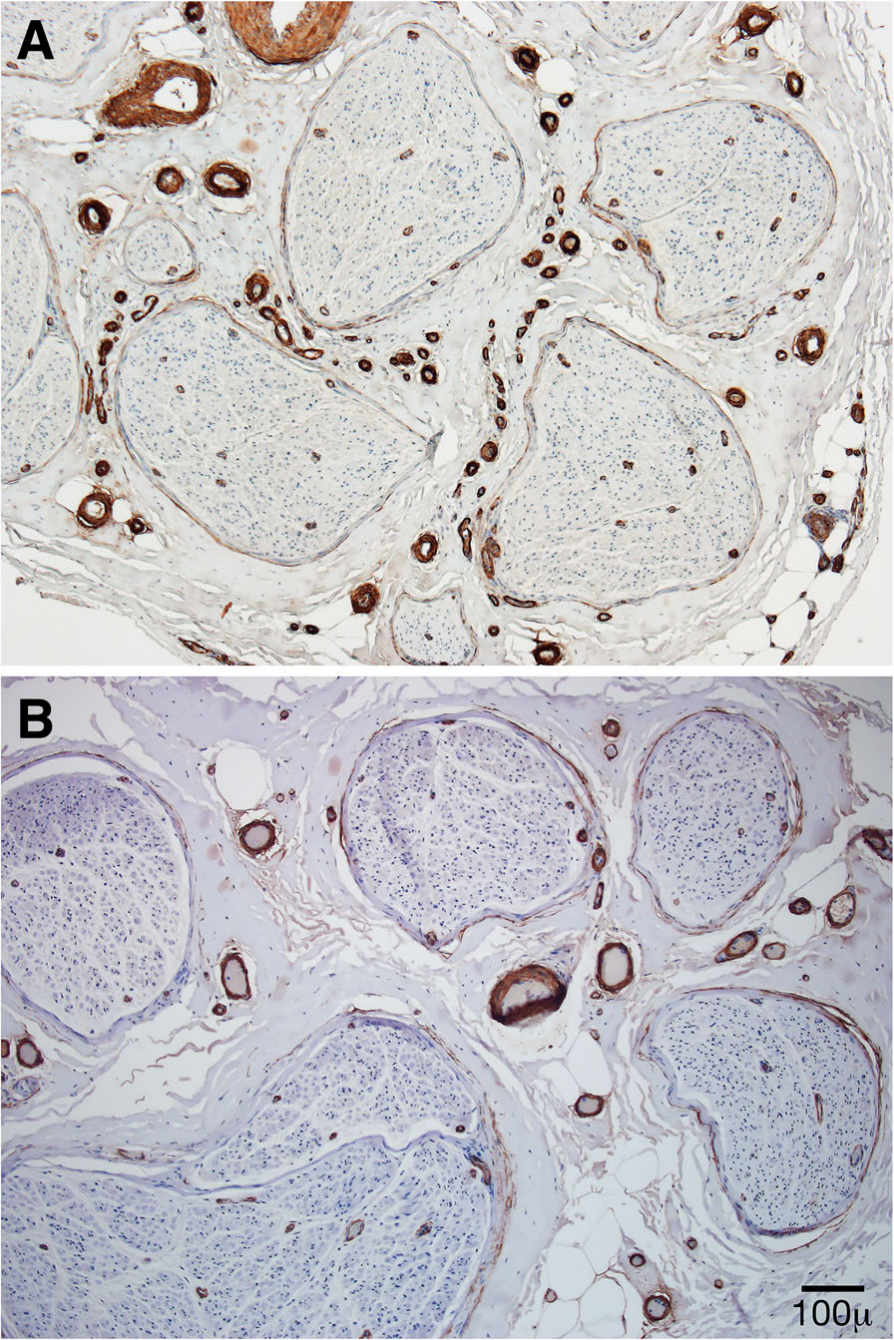
SMACTIN (smooth muscle actin) paraffin cross section from POEMS (**a**) and CIDP (**b**) biopsies. There are increased numbers of small epineurial blood vessels in POEMS (**a**) compared with CIDP (**b**)

**Fig. 6:**
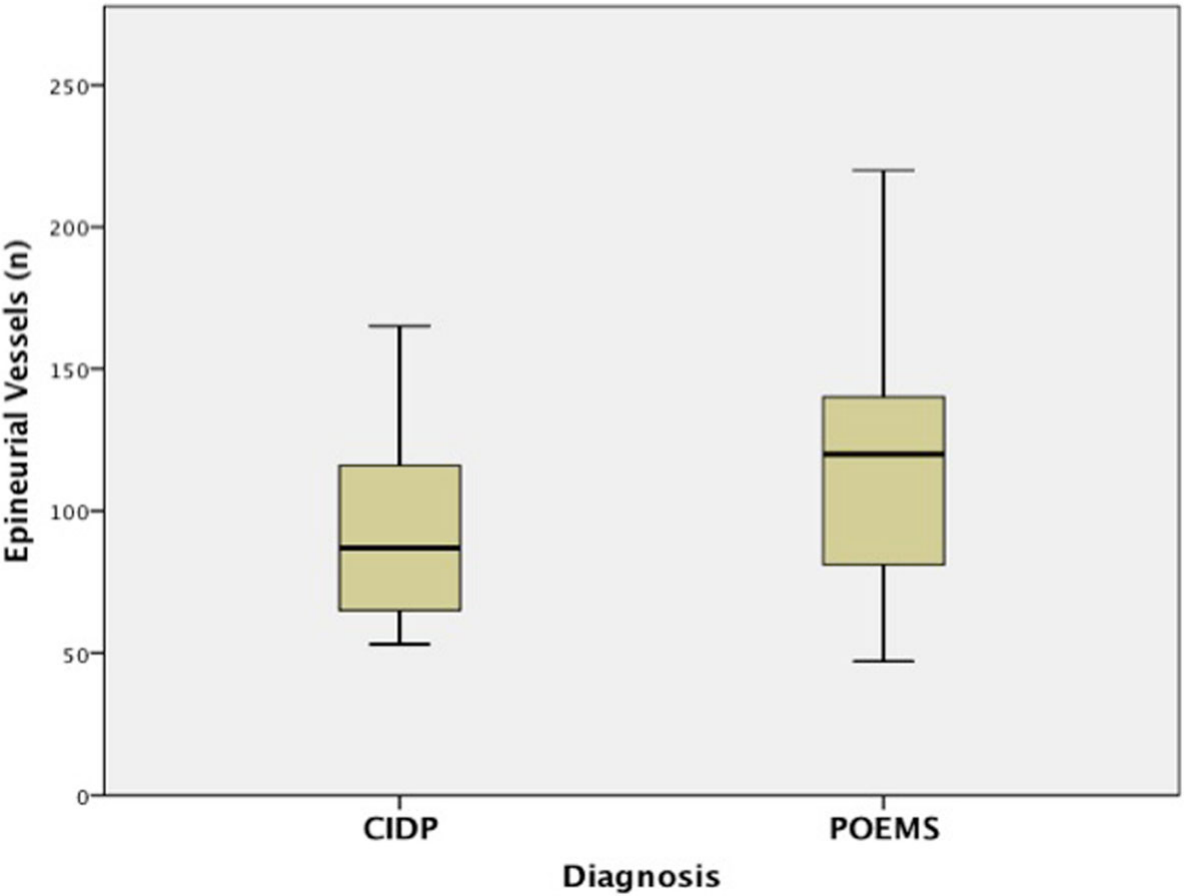
Box-plot graphic of epineurial blood vessel numbers in CIDP compared to POEMS showing that there are significantly more small epineurial blood vessels in POEMS syndrome compared to CIDP. Crossbars represent mean values. Upper and lower borders of the boxes represent the 75^th^ and 25^th^ percentiles, respectively. Upper and lower borders of the whiskers represent the highest and lowest values that are not outliers, respectively

A significantly higher count of small epineurial blood vessels was present in the POEMS group (Fig. 5). The mean number of epineurial blood vessels in CIDP and POEMS were 92 +/− 7 (S.D) and 115 +/− 6 (S.D) (*p* = 0.02), respectively (see Fig. 6). Receiver operating characteristic (ROC) curve revealed a total count of 120 epineurial vessels as the best cutoff (specificity of 77 % and sensitivity of 54 % above 120 suggesting POEMS) to differentiate both conditions (Fig. 6). Counts above this number are more specific but less sensitive to differentiate POEMS from CIDP. Of note, the endoneurial blood vessel counts were not significantly different between both groups.

## Discussion

In this study we found that there are differences in the nerve biopsies of patients with POEMS syndrome and patients with CIDP. Specifically, POEMS syndrome nerve biopsies have higher rates of axonal degeneration, diffuse myelinated nerve fiber loss and increased numbers of small epineurial blood vessels. In contrast, CIDP nerve biopsies demonstrated significantly higher rates of endoneurial inflammation, multifocal myelinated nerve fiber loss and onion bulb formation.

The neuropathy of POEMS Syndrome tends to be severe with progressive impairment of sensory-motor and to lesser degree autonomic nerve fibers with pain being a prominent feature [1, 7, 8]. The pathophysiology of the neuropathy is incompletely understood. One early finding seems to be alteration in sodium and potassium channels present in the nodes of Ranvier and axon [9]. Increased VEGF levels have been associated with the vasculopathy of the vasa nervorum [10, 11]. Other cytokines like IL-1, IL-6, IL-12 and TNF-α as well as growth factors to hepatocytes and fibroblasts have also been found at higher levels [12–14]. An increased level of VEGF but decrease serum level of erythropoietin has been found before [15]. The imbalance between these substances is believed to increase neovascularization and induce functional alterations in the vessel wall (blood-nerve barrier) that lead to increased permeability and edema. The vessel wall alterations are not restricted to the nerve: prior studies have demonstrated similar changes in skin capillaries that improve with chemotherapy treatment [16].

In our study, the finding of increased numbers of small epineurial vessels in the POEMS group supports the idea of a nerve vasculopathy. ROC analysis demonstrated 120 epineurial blood vessels or above as a useful number to suggest POEMS syndrome to differentiate these conditions. POEMS and CIDP cases did not differ significantly in the number of endoneurial blood vessels. This finding is consistent with prior studies, in which the authors suggested that the main change in endoneurial vessels is structural (increased thickness of the basal lamina and narrowed lumen with proliferation of endothelial cells) and not in number of vessels [15, 17]. In fact, one study found a reduced not increased number of endoneurial capillaries with similar morphologic alterations that included narrowing and thrombosis [18]. In our study, we did not see these types of structural changes in the epineurial blood vessels from POEMS biopsies but electron microscopy was not performed and so ultrastructural changes could have been missed.

Electrophysiologically, a demyelinating polyradiculoneuropathy is characteristic of POEMS as well as CIDP. These similarities on electrophysiology are one of the reasons that POEMS syndrome and CIDP are so frequently confused for each other. Prior electrophysiological studies have shown that POEMS syndrome has more axonal loss as evidenced by reduced amplitudes of motor and sensory potentials and greater degrees of fibrillation potentials than does CIDP [7, 19, 20]. Our pathological findings of increased rates of axonal degeneration in POEMS syndrome compared to CIDP provide pathological correlation with these prior electrophysiological studies. We found mild (14/35) and moderate to severe (17/35) degrees of active axonal degeneration in the POEMS cases. Extensive axonal loss (>50 % of fibers) has been reported in smaller case series but this was less common in our study (6 cases of POEMS and 1 of CIDP) [21].

Prior electrophysiology studies have also suggested that the demyelination of POEMS is more uniform than in CIDP affecting all nerve segments including the intermediate ones equally. In contrast, the nerve terminals and proximal nerve segments (nerve roots) are most severely involved pathologically in CIDP [7, 19, 20]. Our study confirmed that both neuropathies have increased rates of segmental demyelination on teased fiber preparations and did not find significant differences in demyelination rates between POEMS syndrome and CIDP. These biopsies are taken from distal cutaneous nerve segments (sural nerves) and this level of the nerve is similarly involved in CIDP and POEMS.

Increased numbers of small epineurial blood vessel formation (neovascularization) from a POEMS nerve biopsy was previously noted in a prior case report [22]. Another case report found evidence of structural derangement of epineurial artery walls with loss of internal elastic membrane [16]. Ultrastructural analysis of POEMS nerve biopsies have found uncompacted myelin lamellae in myelinated fibers [9, 23] which may explain the demyelinating physiology seen in this condition. We did not confirm this finding, as we did not perform electron microscopy on our study.

Our findings of endoneurial inflammation, multifocal myelinated nerve fiber loss and onion bulb formation in our CIDP biopsies are consistent with a chronic inflammatory and demyelinating pathophysiology that have been previously reported in CIDP [2]. Endoneurial inflammation is likely more pathogenic than epineurial inflammation in causing inflammatory demyelination and this is supported by the higher rates of endoneurial inflammation in the CIDP cases. We also found no evidence of chronic repeated inflammatory de- and remyelination in POEMS syndrome biopsies – in other words, we found no evidence of onion-bulb formation in POEMS biopsies as we did in some of the CIDP biopsies.

The main limitation of our study was the retrospective design. The blinding to the diagnosis by the two reviewers was used in an attempt to reduce bias.

## Conclusions

Based on our findings, nerve biopsies can be helpful distinguishing POEMS syndrome from CIDP with POEMS syndrome showing a higher degree of axonal degeneration and more epineurial small blood vessel formation whereas CIDP shows more endoneurial inflammation, multifocal fiber loss and onion-bulb formation. These findings strongly suggest that although POEMS neuropathy and CIDP both present as demyelinating polyradiculoneuropathies fundamentally have different pathophysiologies.

## Abbreviations

CIDP: Chronic inflammatory demyelinating polyradiculoneuropathy
M/F ratio: Male to female ratio
POEMS: Polyneuropathy, organomegaly, endocrinopathy, M protein, and skin changes
ROC: Receiver operating characteristic
SD: Standard deviation
SMACTIN: Smooth muscle actin
